# A human-machine partnered approach for identifying social media signals of elevated traumatic grief in Chicago gang territories

**DOI:** 10.1371/journal.pone.0236625

**Published:** 2020-07-30

**Authors:** Forrest Stuart, Alicia Riley, Hossein Pourreza

**Affiliations:** 1 Department of Sociology, Stanford University, Stanford, California, United States of America; 2 Department of Epidemiology and Biostatistics, University of California, San Francisco, San Francisco, California, United States of America; 3 Research Computing Center, University of Chicago, Chicago, Illinois, United States of America; University of California-Irvine, UNITED STATES

## Abstract

There is a critical need to improve trauma-informed services in structurally marginalized communities impacted by violence and its associated traumatic grief. For community residents, particularly gang-associated youth, repeated exposure to traumatic grief causes serious adverse effects that may include negative health outcomes, delinquency, and future violent offenses. The recent proliferation of digital social media platforms, such as Twitter, provide a novel and largely underutilized resource for responding to these issues, particularly among these difficult-to-reach communities. In this paper, we explore the potential for using a human-machine partnered approach, wherein qualitative fieldwork and domain expertise is combined with a computational linguistic analysis of Twitter content among 18 gang territories/neighborhoods on Chicago’s South Side. We first employ in-depth interviews and observations to identify common patterns by which residents in gang territories/neighborhoods express traumatic grief on social media. We leverage these qualitative findings, supplemented by domain expertise and computational techniques, to gather both traumatic grief- and gang-related tweets from Twitter. We next utilize supervised machine learning to construct a binary classification algorithm to eliminate irrelevant tweets that may have been gathered by our automated query and extraction techniques. Last, we confirm the validity, or ground truth, of our computational findings by enlisting additional domain expertise and further qualitative analyses of the specific traumatic events discussed in our sample of Twitter content. Using this approach, we find that social media provides useful signals for identifying moments of increased collective traumatic grief among residents in gang territories/neighborhoods. This is the first study to leverage Twitter to systematically ground the collective online articulations of traumatic grief in traumatic offline events occurring in violence-impacted communities. The results of this study will be useful for developing more effective tools—including trauma-informed intervention applications—for community organizations, violence prevention initiatives, and other public health efforts.

## Introduction

Exposure to community violence and its associated trauma is a pressing concern. In the United States, an estimated 38% of adolescents have witnessed community violence [1]. These statistics are even more striking in major cities, such as Chicago, where 30% of youth have witnessed a homicide and where, by the age of 15, more than 70% have witnessed at least one serious assault [2]. These numbers are particularly elevated among gang-associated youth and their neighborhood peers, who are overrepresented as victims [3]. Such exposure to violence often results in traumatic grief—defined as the combination of trauma and loss that results from the sudden death of a friend or loved one and that is especially likely in the aftermath of a homicide [4]. Traumatic grief is associated with a range of negative psychological, developmental, educational, and other social outcomes, including involvement in future violence [4, 5]. Unfortunately, youth in hypermarginalized communities affected by gang and gun violence are among the most difficult-to-reach populations in terms of detecting and addressing traumatic grief. New and innovative research and intervention strategies are sorely needed.

This study addresses this need through a mixed-method, human-machine partnered approach that combines qualitative fieldwork, domain expertise, and computational linguistic analyses to pursue two interrelated questions—one empirical and one methodological. Empirically, we investigate how youth and other residents in communities affected by gang violence use social media to articulate and process traumatic grief, particularly in the context of ongoing surveillance and the threat of confrontation by law enforcement, school administrators, and rival gangs. Methodologically, we pursue the question of how to use social media data and computational methods to gather more reliable, scalable, and actionable data on vulnerable, difficult-to-reach, and often “system-avoidant” populations. As Decker and Pyrooz [6: 359] pointedly remind, the search for valid and reliable measures of victimization, particularly among gangs and gang-associated individuals, remains “one of the vexing problems” for criminologists, sociologists, and social scientists more generally. Against this backdrop, we also ask how the integration of qualitative methods and domain expertise can enhance the validity, or “ground truth”, of our computational linguistic analysis. Ground truth is defined as the correspondence between patterns identified by computational techniques and patterns that exist “on the ground” [7–10]. We adapt this term from meteorological research, where it denotes the practice of checking weather forecast models against direct observations of weather conditions occurring “in the real world” (e.g., verifying that a tornado has in fact touched down at the geographic coordinates indicated by models and remote sensing). In our human-machine partnered study of social media, ground truth refers specifically to the correspondence between online signals of traumatic grief and the offline traumatic events that generate those signals.

Applying our collaborative approach to a sample of tweets about traumatic grief associated with 18 different Chicago gang territories/neighborhoods over a five-year period (2012–2016), we find that Twitter contains useful and accurate signals for identifying elevated levels of collective traumatic grief, as measured through the increased frequency of tweets about traumatic grief uploaded from a given territory/neighborhood. The qualitative fieldwork proved highly informative in revealing key processes by which residents used social media to articulate and process traumatic grief. These processes primarily entailed two forms of collective traumatic grief: first, acute grief immediately following loss and, second, grief occurring during anniversaries of loss. The application of computational methods and domain expertise further confirms the generalizability of these two forms of traumatic grief articulation, while also providing evidence for a third, “general,” form of traumatic grief articulation.

Our study makes a number of key contributions to the existing academic literature and provides insights for building community-based interventions that effectively use social media technology. First, our unique study design allows us to better exploit the strengths of both qualitative and computational methodology. The rich, detailed data produced through qualitative, “small data” techniques contribute to the formulation of the initial hypotheses, operative keywords, and analytic strategies for the computational, “big data” component of our study, while also enabling the research team to verify the ground truth of the computational results. Conversely, the computational techniques greatly ensured the generalizability of the qualitative findings. Second, our approach uses “online gang identifiers” as a novel substitute for estimating the spatial context of tweets that otherwise lack location information. Third, this spatialization technique permits us to move beyond the conventional research focus on individual Twitter users as the unit of analysis to analyze online articulations of collective, traumatic grief that occur at the community level. Finally, our approach lays a sociologically grounded foundation for addressing trauma associated with community violence through the use of nonpunitive and trauma-informed programs, thereby reducing the harms (and additional trauma) currently produced by the growing use of social media by law enforcement for arrest and incarceration [11].

To allow for future research and intervention efforts to replicate and build on our approach, we have divided the remainder of the article into five sequential sections. First, we introduce the theoretical background and motivations driving our research, discussing the potential insights that social media data present for better understanding collective traumatic grief as experienced in impoverished, structurally marginalized, and criminalized urban communities, particularly Black, Indigenous, and Latinx communities. Next, we detail the methods and findings from over three years of in-depth, multi-faceted qualitative fieldwork. We draw particular attention to the concrete modes by which community members in gang territories/neighborhoods express traumatic grief online in response to traumatic events occurring offline. We then discuss our methods for the computational linguistic analysis, detailing how qualitative fieldwork produced keywords and strategies for querying and extracting traumatic grief- and gang-related tweets from the general Twitter stream, and for building a supervised machine learning classification system to more accurately create an analytic sample of relevant tweets. Next, we detail our methods for verifying the ground truth of our approach, enlisting domain expertise, content analysis of tweets, and archival searches to verify that online expressions of collective traumatic grief identified by our query, extraction, and classification process do in fact correspond to, or “match”, offline traumatic events. We conclude by presenting the theoretical and methodological implications of our approach, discussing the limitations, ethical considerations, and potential intervention applications of our research.

## Theoretical background and justification: Traumatic grief, gang violence, and digital social media

Public health researchers and professionals have increasingly recognized the detrimental impacts of violence-related traumatic grief on the well-being of communities, and particularly youth [12]. Mental health research demonstrates the deleterious impacts of witnessing the victimization of family and/or close friends [13]. For example, in a study of 245 African-American and Latinx boys living in inner-city Chicago, Gorman-Smith and Tolan [14] found that those individuals who had witnessed a beating, seen someone shot or killed, or had been the victim of a violent act reported elevated levels of anxiety, depression, and aggression. These emotional states are particularly likely to occur during moments of elevated traumatic grief, which may often occur following an acute violence or loss event. Traumatic grief also causes indirect, “collateral” impacts. Exposure to violence impairs the students’ ability to concentrate and to access their stored memories, thereby resulting in poor school performance [15]. Importantly, traumatic grief is a *collective*, community-wide phenomenon. Local residents may not even be required to directly or personally experience violence to feel its traumatic effects. Sharkey [16] found that students who took a cognitive test in the days following a homicide in their neighborhood scored lower than their peers who took the test at a different time. In the aggregate, neighborhood violence accounts for almost half of the association between neighborhood disadvantage and high school graduation among boys and almost all of the association among girls [17].

Given the strong link between gang affiliation and exposure to homicide, gang-associated youth and their peers are likely to experience the highest levels of traumatic grief in their communities. Although young people typically form, join, and associate with gangs for safety, this association actually *increases* their risk of victimization [18]. Unfortunately, in terms of public health and mental health interventions, gang-associated youth, peers, and fellow neighborhood residents are among the most difficult-to-reach and underserved populations. Unlike those individuals experiencing traumatic grief in better-resourced neighborhoods, structurally disadvantaged minority communities—particularly those characterized by a high proportion of gang-associated residents—are more likely to have had previous harmful contact with the criminal justice system and to have experienced elevated levels of coercive state surveillance [19–23]. Such contact results in system avoidance—defined as “the practice of individuals avoiding institutions that keep formal records (i.e., enter them in the system) and therefore heighten the risk of surveillance and apprehension” [19]. System-avoidant populations are less likely to engage with employment institutions (e.g., workplaces), financial institutions (e.g., banks), educational institutions (e.g., schools), and—most important for the current study of traumatic grief—health institutions (e.g., hospitals, counselors, and mental health professionals). In fact, individuals experiencing criminalization and surveillance have 33% higher odds of not obtaining medical care, even when they need it [19].

Fortunately, the proliferation of social media and the accompanying development of computational linguistic analyses has created new opportunities for detecting and addressing mental health needs, particularly among system-avoidant populations who are far less likely to proactively seek out mental health care or similar assistance. Twitter is especially useful in this regard. Twitter is a free social networking and microblogging service that enables users to send and read each other’s “tweets”—short, 280-character messages. The service currently has more than 126 million daily users and processes approximately 500 million tweets per day [24]. The range of Twitter’s mental health applications include, for example, predicting depression [25], identifying responses to high profile suicides [26], and monitoring socialization surrounding eating disorders [27]. Twitter’s large number of users and massive corpus of content represent great opportunities for data and text mining [28]. By far, the greatest advantage offered by Twitter analysis is its temporal immediacy. Twitter activity can be obtained and analyzed almost instantaneously and via automated processes.

Twitter is even more useful given the composition of its user population. Today, Black teens and millennials (ages 18–39 years) continue to rank as the most frequent users of Twitter, engaging with the platform at approximately double the average rate of that of all Twitter users [29, 30]. However, despite these continued usage trends, and despite the growth of #BlackTwitter over the last half decade [31, 32], there is a dearth of public and mental health research focusing specifically on Twitter use by Black populations. Although there is similarly scant literature on traumatic grief among gang-associated youth and their peers and even less research on how this grief manifests online, a small handful of pioneering studies suggests that gang-associated youth may indeed articulate their experiences of traumatic grief online [33, 34]. Most notable is a series of research articles examining the Twitter activity of a now-deceased gang-affiliated Black youth in Chicago [35, 36]. For example, Patton et al. [33] find that a substantial volume of the individual’s tweets appear to discuss death, loss, and grief.

The phenomenon of online “grief work,” or mourning the death of a loved one on social media, is not unique to gang-associated youth and their peers [4]; however, what is distinct is the heightened significance and potential consequences of public, online grief work among this particular population. First, given the hypermasculine norms of street culture that disincentivize displays of vulnerability and weakness [37], and the experiences of surveillance and criminalization that produce elevated system avoidance, online platforms, such as Twitter, may represent one of the few acceptable outlets for expressing difficult emotions. Second, given its elevated importance, the articulation of traumatic grief online may foreshadow, and potentially even predict, violence both online and offline. Preliminary evidence suggests the possibility of such a relationship. Patton et al. [34] find that tweets expressing loss are predictive of tweets expressing aggression. In the day following tweets about loss, there is a 13% increase in aggressive tweets. This number climbs to 21% in the second day, before falling to statistically insignificant levels in the following days. Although no research to date has determined the precise association between aggressive social media content and offline violence, one existing study has shown that, uploaded under certain conditions, a small number of aggressive social media statements may catalyze retaliatory violence [38].

The few existing studies of online grief work among gang-associated youth and their peers represent an important first step toward understanding and addressing a greatly-overlooked and misunderstood phenomenon. However, previous research is characterized by several limitations, which the current study seeks to overcome. First, prior studies have had difficulty verifying the ground truth of the inferences drawn from online content. By focusing primarily on decontextualized and disembodied tweets, and not integrating offline fieldwork alongside the authors of those tweets, prior studies are limited in their ability to determine the offline contexts, meanings, and motives associated with those particular tweets. Existing approaches similarly lack the methods necessary for verifying whether particular online expressions of traumatic grief actually correspond to offline events. Second, current research has been limited to studying tweets about traumatic grief at the individual level, and among individual users. This limitation is due primarily to the fact that a vast majority of tweets (over 99%) lack location information [39], which makes neighborhood-level data collection and analysis far more difficult. However, as the literature shows, traumatic grief is often a community-wide phenomenon. As such, it is imperative to identify and examine collective expressions of grief that occur within extended social networks and/or at the neighborhood scale. These limitations are not merely academic; they have implications for building effective public health interventions and ensuring the ethical treatment of long-maligned communities of color.

To overcome the limitations of previous research, we make good on boyd and Crawford’s [40] provocation that “in this computational turn, it is increasingly important to recognize the value of ‘small data’” (670)—that is, the data produced through deep, purposeful, in-depth field methods, such as ethnographic observations and qualitative interviews. As Abreu and Acker [9] argue, the most important characteristic of these small data methods is their capacity to provide *context* surrounding online content, such as tweets, by collecting data not only about the online content but also, and perhaps most importantly, about other events in the individuals’ and the communities’ daily offline lives. In our case, the immediate empirical phenomenon of interest is the online articulation of traumatic grief. However, offline context is vital for understanding such online activity. Integrating small data methods thus enhances ground truth, allowing us to discern the relationship between such online articulations and their associated offline events (e.g., instances of homicide and traumatic loss). This approach similarly allows us to more accurately uncover the social meanings and offline implications of such online articulations. As with all social media data, tweets provide ample room for misinterpretation. Among outsiders, particularly among law enforcement and other surveillance institutions, misinterpretation has already produced serious documented harms, including increased punitive surveillance, expanded criminal justice entanglements, and even erroneous convictions [38, 41, 42]. Furthermore, by shifting focus to the community level (i.e., analyzing *collective* Twitter activity), our method reduces the dangers that this research will be used maliciously to target individual Twitter users.

## Qualitative fieldwork: Understanding traumatic grief on Twitter

How do gang-associated youth, their peers, and other local residents articulate and process traumatic grief on social media? We began pursuing this question as part of a three-year, community-engaged, qualitative research project called the South Side Youth Violence Prevention Project (SSYVPP). The SSYVPP’s aim was to investigate the broader role of social media in the social organization of gangs, gang violence, trauma, and youth life on Chicago’s South Side. Through a multiyear partnership with two community-based organizations, research team members collected the following data: (a) four months of weekly observations of youth participants (ages 14–17 years) in a trauma-informed, violence-reduction after school program developed and codirected by SSYVPP; (b) 120 formal, in-depth interviews with neighborhood youth (ages 15–25 years); (c) 24 months of daily observations of approximately 30 youth (ages 15–28 years) associated with a South Side gang faction known as the “Corner Boys,” or “CBE” for short; (d) 12 months of daily observations of outreach workers, victim advocates, and directors of a Chicago violence prevention organization. Fieldwork conducted for this study was approved by the University of Chicago Institutional Review Board (#14–0829). Researchers obtained consent in both oral and written forms. One of the community partners—a city-wide non-profit organization specializing in youth safety and violence prevention—served as our community advisory board, assisted in study design, and provided input on interview procedures and questions, as well as interpretation of findings.

The qualitative fieldwork identified two primary practices by which gang-associated youth and their communities articulate traumatic grief on Twitter: (1) expressing acute grief immediately following the loss of a friend or loved one and (2) expressing grief on the anniversary of a significant loss or other important dates related to the deceased (e.g., birthdays). The fieldwork also revealed important offline, contextual factors that accompany both of these uses of social media. The most intense way in which communities collectively articulate traumatic grief online—namely, expressions of acute grief—occurs in the hours and days immediately following loss. For example, consider the Twitter activity following the murder of a young man named Javon (known locally as “J-Boy”). During the fieldwork period, Javon was the victim of a drive-by shooting. Javon was not formally gang-associated, but the deadly assault occurred while he was walking with his best friend, who is named Junior and was affiliated with the CBE gang faction. According to Junior, who survived the attack, a car full of rivals were driving past when they recognized him and opened fire on both of the young men. When the news of Javon’s death began reaching his peers and neighbors later that day, they immediately began expressing their emotions on social media. On Twitter, peers and neighbors uploaded photos of the young man, shared favorite memories, and communicated their sadness. The following tweets were authored by different users in the days following Javon’s death:

- RIP J-Boy
- Can’t sleep Wish J-Boy was still here
- I lost another one Rest Up Javon CBE keep ya head up
- My boy Javon just died
- R.I.P J-Boy #CBE
- LONG LIVE JAVON

The second primary mode of articulating traumatic grief is the expression of grief corresponding to the anniversary of a loss. This mode consists of tweets intended to memorialize the birthday or date of death of the deceased. In Chicago, residents refer to these anniversaries as “dead homie days” and use these occasions to publicly convene to engage in block parties, cook-outs, candlelight vigils, and other acts of remembrance. For example, following the shooting death of a young man named Benzie, his friends, neighbors, and fellow CBE members informally renamed the neighborhood the “Benzie Block”, began referring to CBE as either the “Benzie Block” or the “Benzie Gang”, and declared both Benzie’s birthday and his date of death to be “Benzie Day.” Twice a year, on these dates, neighborhood residents gather at the site of Benzie’s death to memorialize him. On Twitter, neighborhood residents share their memories of the young man, express their emotions over his loss, and spread invitations for others to attend the day’s memorial:

- Happy BENZIE day to all my #CBE brothers
- RIP Benzie Its Benzie Day! #CBE
- Happy bday Big Benzie #RIP
- Turn up for Benzie Day S/O CBE

Some neighborhood residents more frequently engage in this form of expression. This form of expression occurs among those who were particularly close to the deceased or who were most impacted by the death. Rather than wait until a dead homie day, these residents use Twitter to express their traumatic grief on a weekly or even a daily basis. A young woman named Bree, one of Benzie’s best friends since childhood, is one such example. Throughout the fieldwork period, Bree authored a tweet about traumatic grief memorializing Benzie every morning. She described this practice in an interview:

I’ll try every day at 9:14 AM. I always track his—that’s his date [of death; September 14th] and at 9:14 AM, I want to always put up something about him. I always save 9:14 AM for Benzie. … They [the tweets] say “Benzie Block, we miss you,” and stuff like that.

Interviews and observations conducted with residents immediately following traumatic loss and during anniversary periods provided critical contextual insights regarding the social meanings of such online expressions. Expressions of traumatic grief on Twitter constitute a collective practice of solidarity and support. Community members relayed that such tweets are not simply a way for them to articulate their own pain and engage in mourning; rather, these tweets are a vital means of communicating their continued love of, and commitment to, their family, friends, and neighbors. One of Javon’s childhood friends articulated how he felt when he saw the large number of neighborhood residents memorializing Javon on Twitter:

I could see [on Twitter] I ain’t alone. We all lost something when he died. Even some people I didn’t know was even close to him. It’s crazy. We kinda, like, got a bond over that now. He touched a lot of different people.

Among residents, expressions of grief online—both immediately following a loss and during anniversary periods—have become a sort of litmus test that residents use to determine the depth of others’ love and sympathy for the deceased and, by extension, for themselves. As another one of Javon’s childhood friends relayed:

You could see who really messed with [cared about] him [the deceased] by what they put up [on Twitter]. That’s how I know who *really* got *my* back. When they show Javon love, they really showing me love too. They know I’m over here messed up. If they don’t got time to show *him* love, then I ain’t got time for *them*!

In the qualitative fieldwork, recurring linguistic patterns and associated social meanings throughout traumatic grief tweets were found. First, these tweets frequently contained three particular keywords to express loss of and fondness for the deceased. These were the following: “RIP,” “Rest Up,” and “Long Live.” Unlike other keywords that residents use in times of distress, these keywords are highly specific to situations in which a loved one, peer, or neighbor has died. These keywords are highly unlikely to be used in other contexts. This finding confirms previous studies of Twitter use among urban poor youth; these studies identified these phrases as the top keywords for conveying feelings of loss [33, 35, 43]. Second, traumatic grief tweets often mention the local gang faction, which is referenced either by its full formal name (e.g., Corner Boys), an acronym (e.g., CBE), or a variant memorializing a deceased member (e.g., Benzie Gang). These tweets also frequently reference the informal name of the gang territory/neighborhood, particularly when the area has been renamed in honor of a deceased resident (e.g., Benzie Block). Again, qualitative fieldwork identified multiple sources for this pattern. Given the deep social significance of traumatic grief tweets—particularly their capacity to convey the collective support for peers, neighbors, and the community at large—gang-associated youth are among the most prolific authors of such tweets in their neighborhoods. For residents who are not affiliated with gangs, mentioning the name of the local gang faction in a tweet often constitutes a means for conveying support or at least a *lack* of animosity toward the local faction. For example, residents seek to communicate that they will not call the police on the gang members or interfere with their illicit economic activity. For some residents in the fieldwork communities, the local gang faction name has also become interchangeable with, or even a substitute for, the official name of the neighborhood. Thus, both residents associated with gangs *and* residents not associated with gangs primarily refer to their neighborhood in gang terms—for example, as Benzie Block. In fact, such self-identification has become a premier means for longtime residents to convey their community pride and cement their elevated status as a “local.” These informal neighborhood labels frequently appear on custom silk-screened shirts and throughout social media activity, including Twitter handles, user descriptions, and other content. As previous research has consistently shown, the local residents’ outward identification with, and support for, local gang factions is common in violent and/or gang-controlled neighborhoods [44, 45].

These qualitative findings shed much needed empirical light on the ways in which gang-associated youth and their communities articulate traumatic grief on Twitter. However, as is the case with many qualitative studies, it is difficult to assess the generalizability of these findings beyond the sample population. Although the qualitative data collection was extensive and included residents from multiple neighborhoods and gang territories, several important questions remain: To what extent do residents in *other* Chicago neighborhoods and gang territories collectively articulate traumatic grief on Twitter? How common is this practice, generally, and how prevalent are the two modes of grieving, specifically? In the following section, we detail how we examined these questions by developing a computational linguistic analysis of the larger, daily Twitter stream.

## Computational analyses: Using Twitter to identify moments of collective elevated traumatic grief

**Fig 1:**
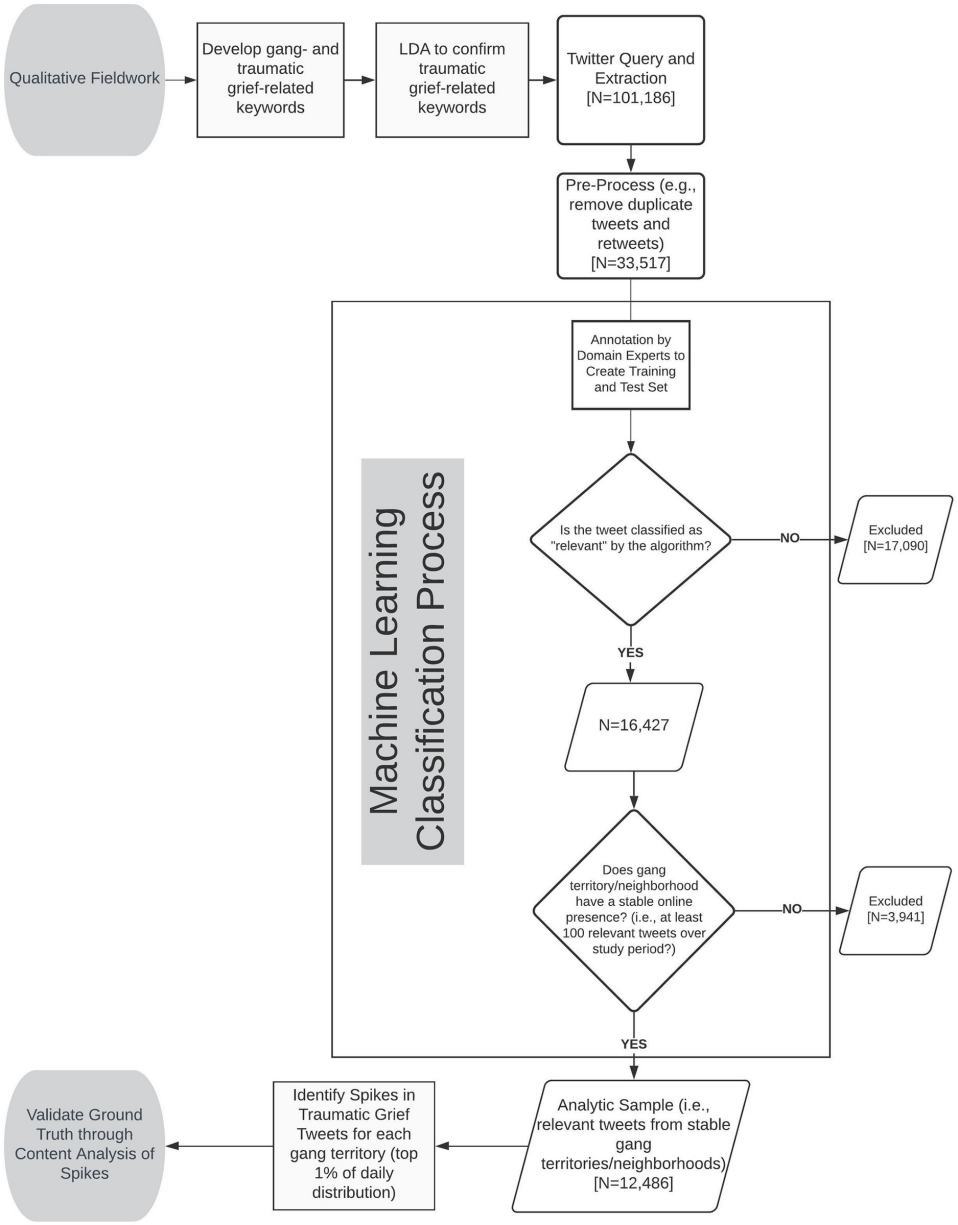
Workflow for qualitative fieldwork, machine learning classification, and content analysis.

Informed by our qualitative fieldwork, we sought to develop a computational approach that leverages Twitter data to observe our phenomenon of interest—moments of collective traumatic grief occurring in gang territory/neighborhoods. One of the primary challenges in using social media data to identify offline events and mental states is that social media data contain high levels of noise. Given the vast and varied uses and users of Twitter, even the most careful search of the Twitter stream can return a large amount of irrelevant or unrelated content. This dilemma is compounded by the use of automated search methods, which often lack the capacity to process nuanced meanings and context-laden content, and run the risk of creating “automation bias” [46]. To address this challenge—to reduce the noise contained in the sample of tweets used in our analysis—we developed sequential steps for preprocessing and a machine learning classification to distinguish relevant from irrelevant tweets. The following subsections detail our method. Fig 1 provides an overview of the workflow.

### Keywords for Twitter query and extraction

The first step in extracting only gang- and traumatic grief-related tweets from the massive Twitter stream was to generate a list of keywords to use as search terms to query Twitter. Given that keywords selection can introduce error and bias results, it is imperative to carefully evaluate the potential keywords. Our selection of keywords was greatly enhanced by qualitative fieldwork. The selection of keywords began with gang-related keywords. First, to assemble a sample of gang territories/neighborhoods on Chicago’s South Side, we drew on the Chicago Police Department’s list of gang factions (and their territories), which is published in the latest Chicago Crime Commission *Gang Book* [47]. Recent criminological research supports the robustness, reliability, and validity of police and other agency reports of gang crime, gang prevalence, and gang membership [6]. Decker and Pyrooz [6: 371] contend that “those who argue that gang measures reported by the police are political, haphazard, or unreliable, now face the burden of documenting that such claims are true.” Nevertheless, given the ethical implications of our study for criminalized, surveilled, and vulnerable populations, we sought additional verification of the gang data delineated in the *Gang Book*. We consulted with local organizations, youth residents, and other community stakeholders about our list of local gangs and gang territories. Together, we constructed a sample of 57 unique gang territories/neighborhoods. We selected these 57 areas based on the stability of the gang faction and the geographic diversity in our sample. In a later step, we further narrowed our sample to the 18 gang territories/neighborhoods with a stable online presence (which we defined as having at least 100 relevant tweets in our study period).

Second, we determined the “online gang identifiers” for each gang territory/neighborhood in our sample by consulting the qualitative data, interviewing gang-associated youth, and conducting online searches. These keywords consisted of the formal gang faction names, acronyms, informal names based on online grieving practices, associated hashtags, and other relevant words and phrases that residents associated with gangs or not associated with gangs use to signal their affiliation with, or proximity to, particular gang territories and neighborhoods.

Our use of online gang identifiers allows us to approximate each tweet’s spatial context—that is, the physical location of the events discussed and people referenced in the tweet. This represents a novel technical advance, and it allows us to group tweets that refer to a collective experience. One of the main obstacles preventing neighborhood-level analyses using Twitter data is the lack of systematic locational data provided by users, who overwhelmingly turn off location services. Previous studies report that only 0.85% of all tweets are geotagged [39]. As a result, although computational linguistic analyses may be able to ascertain users’ potential sentiments and mental states, it is difficult to discern the precise location of the events associated with these sentiments and mental states. Some researchers have addressed this challenge by restricting their studies to only incorporate geotagged tweets or those authored by known subjects; however, this leads to severely diminished sample sizes [33, 35]. This approach is particularly problematic when research draws on content from gang-associated and violence-involved Twitter users, who are even less likely than the general public to turn on location services. Rather than omit nongeotagged tweets from our study, our methodological approach employs the Twitter users’ self-identification with, or mention of, a particular gang faction and a territory as a substitute measure of spatial context. Because gang factions inhabit and control particular neighborhood streets and blocks as part of their turf [48–50], those who self-identify with a given gang on Twitter via online gang identifiers are likely to live in, spend considerable time in, or, at minimum, have meaningful social ties with those who inhabit that gang’s designated space. Of course, it is plausible that some users may upload tweets containing such online gang identifiers while physically located outside of the gang territory/neighborhood—at school or work, for example. Even in such cases, these users are nonetheless actively expressing their identification and affiliation with a distinct gang territory/neighborhood. Furthermore, whatever their exact location at the time of their post, such tweets reference or are directed toward the specific events and people within that particular gang territory/neighborhood. Given that our analysis is ultimately concerned with identifying online signals of offline events experienced collectively, the use of online gang identifiers is a systematic and specific method for identifying tweets expressing traumatic grief that reference the same local context.

In total, our process produced 340 online gang identifiers (search terms that uniquely identify one gang faction), with an average of 4 identifiers per gang faction in the sample. We excluded gang identifiers that consisted primarily of numbers (e.g., “#700,” or “059”) because of the numbers’ lack of specificity and their elevated probability of returning irrelevant tweets (tweets where the identifier appears but is used to refer to something other than the phenomenon of interest). (Note that this is an observational study. In compliance with Twitter user agreements, our computational analysis draws on retrospective, publicly available Twitter data. To minimize any threat to personal privacy, we have obscured any mention of user handles in our presentation of findings. We also refer to gang factions in the computational analysis and findings by number rather than by name).

Our selection of traumatic grief-related keywords was similarly enhanced by qualitative fieldwork and additional computational techniques. First, ethnographic fieldwork, interviews, and consultations with local organizational partners revealed three keyword phrases that are most likely to be utilized to express traumatic grief: “RIP,” “Long Live,” and “Rest Up.” Second, we evaluated the appropriateness of these keyword phrases by performing Latent Dirichlet Allocation (LDA) topic modeling on all gang-related tweets collected in the month following the murder of Javon (detailed above in Section 3) [51]. LDA is a standard method for automatically discovering the topics and sets of topics contained in a corpus of text. LDA allows researchers to assess which words and groups of words are most likely to be associated with a given topic—in our case, traumatic grief. Our domain expert annotators reviewed the top keywords that resulted from the LDA topic modeling. Excluding the various derivations of the names of the victim, gang, and gang territory/neighborhood, 5 of the 6 most frequently tweeted traumatic grief-related words reflected at least one of those that we previously identified in our qualitative fieldwork. In order of frequency, these keywords were the following (with their respective rank noted in parentheses): RIP (1), Live (3), Long (4), Peace (5), and Rest (6). Our selection of traumatic grief-related keywords is further confirmed by recent studies of the expressions of loss and grief by gang-associated youth on Twitter [36, 41, 52]. Much of the analytic strength of our three traumatic grief keywords is rooted in their specificity. These keywords are highly specific to situations in which a loved one, friend or neighbor has died. It is extremely rare that these keywords are used to express emotions for situations *other* than this type of traumatic loss.

### Twitter query and extraction

After finalizing our selection of traumatic grief keywords and gang keywords for our sample of Chicago gang factions, we collected all tweets from January 1, 2012, to December 31, 2016, that contained a combination of at least one traumatic grief keyword and one gang keyword. To do so, we used the Premium Twitter Application Programming Interface (API). The API is a set of routines, protocols, and tools that allow for the creation of applications for accessing and downloading the data of an operating system or platform, which in this case, was Twitter. To maximize our potential returns, we also collected all tweets that contained a combination of at least one traumatic grief keyword (e.g., “RIP”) and one gang keyword appended by the suffix “k.” This suffix is short for “killer” and is used to simultaneously identify one’s own gang faction and one’s rival gang faction.

#### Pre-processing

Before any preprocessing, the query returned 101,186 total tweets. We began preprocessing the data by using several methods. First, given our underlying interests in traumatic grief and coping as a community-level phenomenon, we excluded duplicate tweets sent from the same Twitter user on the same day. Duplicate tweets from the same user were recorded as a single tweet. Second, we excluded any tweets that did not contain at least one grief-related keyword and one gang-related keyword in the tweet body or “text field.” Third, we excluded tweets whose text field mentioned another Twitter user whose handle contained one of the traumatic grief keywords. Fourth, in order to prevent the skewing of our analysis as a result of retweets, we excluded all retweets. After performing a sequence of string processing operations, our refined dataset contained a total of 33,517 tweets, of which 30,314 tweets contained the word “RIP”, and 3,165 tweets contained “Rest Up” and/or “Long Live”. Finally, after the annotation by human experts, to allow the use of our classification algorithm on tweets assigned to any gang faction in the dataset, we rendered each tweet nongang specific. To do so, we masked gang identifiers, urls, email addresses, phone numbers, and mentions by replacing them with _IDENTIFIER_, _URL_, _EMAIL_, etc. We also converted the text to lowercase before masking (to ensure an exact match of gang identifiers), replaced punctuations with spaces, removed tokens of size 2 or shorter, replaced 3 or more occurrences of the same character with one (e.g., looool became lol), and lemmatized the text. To allow human annotators access to the full text, we masked online gang identifiers after the hand-coding.

### Hand-coding and classification of relevant tweets

Studying traumatic grief associated with gang violence on Twitter is difficult because the traumatic grief keywords and the gang keywords frequently appear together in tweets that are not related to traumatic grief or gangs in Chicago. We refer to these tweets that fail to use the keywords in relation to the phenomenon of interest as “irrelevant tweets.” To classify relevant and irrelevant returns from Twitter and thus improve our ability to track traumatic grief online, we developed the following machine learning approach.

We used supervised learning to construct a binary classification algorithm that sorted the tweets returned by the query process into two groups: relevant or irrelevant. First, hand-coding annotation was performed by two domain expert members of the research team, both of whom are PhD sociologists with over 18 combined years of experience in researching and designing interventions related to urban violence, gangs, and trauma. These annotators were also the research team codirectors of the SSYVPP after-school program and lead researchers on the qualitative data collection process. Drawing from their expertise and insights gained during the qualitative fieldwork, the annotators mutually created a set of rules to assess whether a given tweet was indeed “relevant” or “irrelevant.” The rules were as follows:

- Tweets that contain at least one traumatic grief keyword *and* at least one gang keyword were considered to be indicative of *relevant* tweets.
- Tweets that discuss events occurring outside of the city of Chicago, discuss the deaths of celebrities or other famous individuals, lack one of the trauma grief keywords or online gang identifiers, or use a keyword or identifier primarily in non-trauma- or non-gang-related ways were considered to be indicative of *irrelevant* tweets.

Following these mutually agreed upon rules, the annotators coded a randomly selected subset of 2,219 tweets from 2016 to create a training set for the purpose of classification. Each annotator independently reviewed the content of each tweet and determined whether it was relevant or irrelevant. The raters had near perfect agreement—the interrater reliability metric, Cohen’s [53] kappa, was found to be .95 (where 1.0 corresponds to 100% agreement between annotators). The few discrepancies were resolved through mutual discussion. Of the training set, this process identified 861 total tweets as relevant and 1,358 tweets as irrelevant. Alternative classification and validation techniques support our methodological approach (see S1 Appendix).

After preprocessing and classifying relevant tweets (see S1 Appendix), we found that only 18 gang factions had a stable online presence over our 5-year study period (defined as at least 100 relevant tweets over the study period), and therefore, we further reduced our sample. For this reduced sample of 18 gangs with a stable online presence, our procedure identified a total of 12,486 traumatic grief-related tweets, which constitutes our analytic sample.

## Matching elevated traumatic grief on Twitter to offline events

### Identifying spikes in collective traumatic grief on Twitter

Up to this point, we have described how our novel approach uses Twitter data to observe, almost in real time, the latent phenomenon of collective traumatic grief among gang-associated youth. We are now positioned to evaluate the effectiveness of this approach: we will assess how well the online *signals* of traumatic grief on Twitter, as identified by our approach, correspond to, or “match”, real-world, offline *events*. In this section, we present descriptive findings that suggest our approach provides a relatively high match rate and satisfies the need to ensure ground truth.

**Fig 2:**
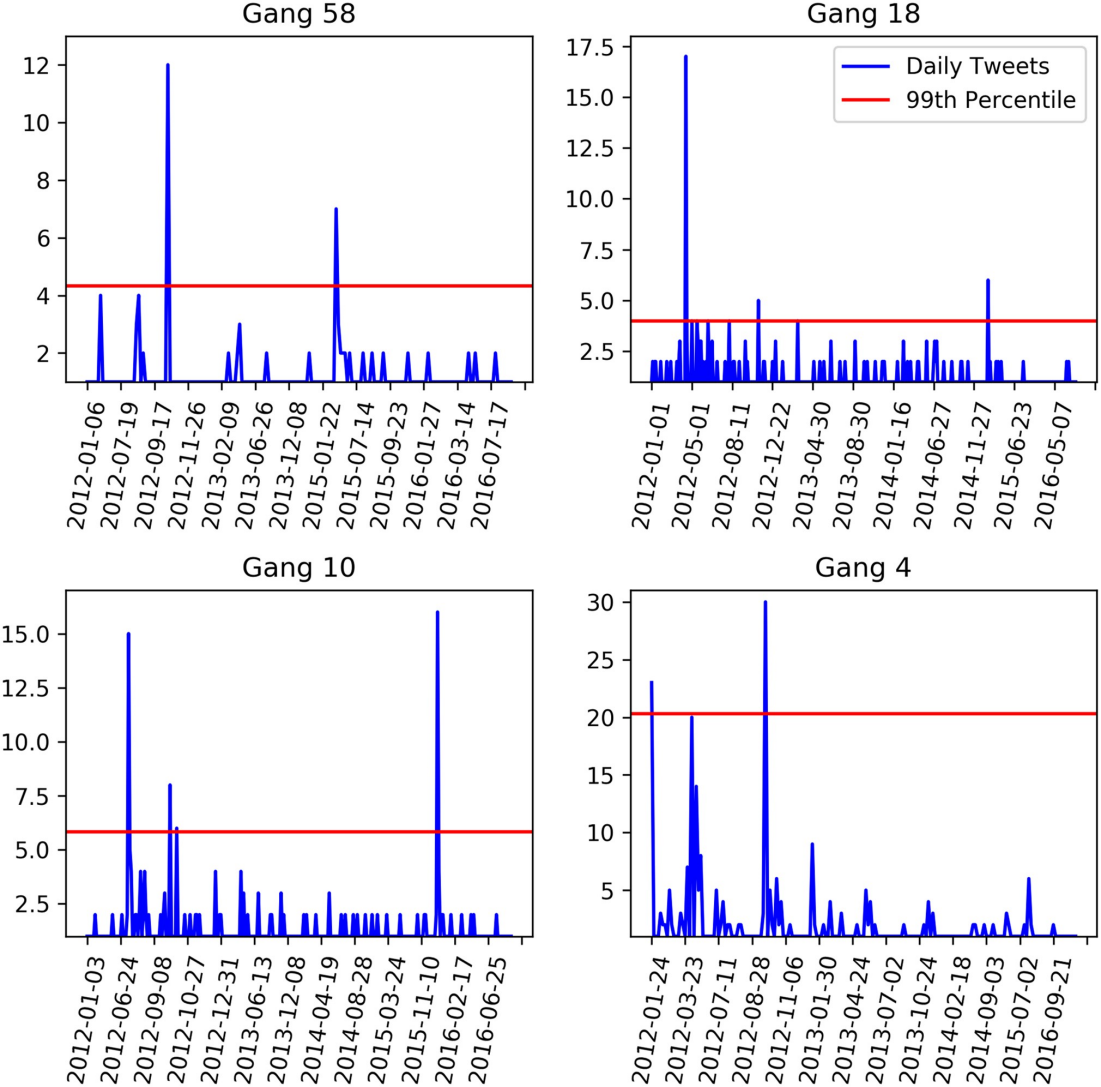
Standardized spikes for four sample gang territories/neighborhoods (2012–2016).

Recall that our underlying intention is to identify specific calendar days that are characterized by elevated levels of collective traumatic grief associated with a particular gang territory/neighborhood. For the 18 gang territories/neighborhoods in our analytic sample, the daily average of traumatic grief-related tweets was 0.35. Despite this relatively low daily average, many days were characterized by noticeable increases, or “spikes,” in traumatic grief-related tweets, reaching as high as 155 tweets in a single day. Given our concern with the most acutely and collectively experienced moments of elevated traumatic grief, and guided by our theoretical background, we focused our analysis on the top 1% of such single-day spikes for each gang faction in the subsample. This approach produced a total of 114 single-day spikes, with an average of 12.53 tweets per spike. To ensure that the metric is comparable across gang territories/neighborhoods, we identify spikes relative to the baseline for each particular gang territory/neighborhood. This standardization process is necessary to make comparisons across various territories/neighborhoods characterized by relatively different sized populations and tweet frequencies. Standardization is also accomplished by excluding retweets and collapsing identical tweets authored by the same user on the same day into a single tweet. We do not exclude or collapse when users author the same tweet text on the same day, because such online expressions are key instances of collective expressions of traumatic grief. Fig 2 provides examples of standardized spikes in four of the gang territories/neighborhoods in the sample.

### Evaluating ground truth: Description of spikes in collective traumatic grief tweets

To understand the extent to which expressions of collective traumatic grief on Twitter correspond to, or match, offline traumatic events and mental states, under the supervision of domain expert annotators, the research team examined the text field of every tweet that comprised each spike in Twitter activity to identify the names of the specific individual subjects to whom the “RIP,” “Long Live,” and “Rest Up” keywords were applied. By consulting the Twitter users’ profiles, using the Twitter search function, and conducting Google searches, the research team identified the full legal name, date of death, and birthdate (when possible) of each subject. Team members triangulated this information by using a variety of archival sources, including homicide victim databases provided by the *Chicago Sun-Times*, *Chicago Tribune* and other local publications.

For example, for one of the gang factions in the subsample (Gang 6), our process identified the following five-tweet spike (with each tweet authored by a unique user) on March 9, 2012.

- This [GANG 6]! rip lil Josh!
- another [GANG 6] soldier down rip lil josh
- @[Handle Obscured] 40 BOI–[GANG 6] YOU WILL ALWAYS BE LOVED #LILJOSH
- REST UP [GANG 6] LIL JOSH
- REST IN PEACE [GANG 6] LIL JOSH

In this example, our subject identification and triangulation procedure determined that the five tweets causing the spike on March 9, 2012, referred specifically to the death of 16-year-old Joshua Williams (also known as “Lil Josh”), who was killed the day prior, March 8, 2012. Williams’ death took place squarely within the approximately seven square-block territory controlled by Gang 6.

This analysis was repeated for all 114 spikes of elevated traumatic grief among the subsample. We defined a “positive match” as a significant spike for which we were able to confirm correspondence with a death. Of the 114 total moments of elevated collective traumatic grief identified by our computational techniques, 91 were determined to be positive matches, yielding a mean match rate of 83.4%, with a median match rate of 91.3% (see Table 1). Among the subsample, 8 gang factions yielded a 100% match rate with offline events, while 11 gang factions yielded match rates of 90% or above. Although the universe of offline trauma is unknown, we find that traumatic grief-related Twitter spikes detected by our method correspond to offline events with an impressively high match rate. In short, our procedure successfully identified moments of elevated collective traumatic grief, as indicated by sudden and large increases in the daily frequency of grief-related tweets.

**Table 1:** Elevated collective traumatic grief by gang territory/neighborhood.

Gang #	Tweets	Daily Average	Daily Spikes in Elevated Traumatic Grief	Number of Matches	Match Rate (%)
5	970	0.53	10	9	90
6	177	0.10	2	2	100
7	1157	0.63	12	11	91.7
8	2699	1.48	14	7	50
10	437	0.24	4	2	50
15	994	0.55	6	6	100
16	796	0.44	10	7	70
17	334	0.18	6	4	66.7
24	1083	0.59	11	10	90.9
26	370	0.20	3	3	100
27	162	0.09	2	2	100
36	182	0.10	4	4	100
37	1015	0.56	7	6	85.7
43	1157	0.06	11	9	81.8
45	229	0.13	4	4	100
49	151	0.08	2	2	100
54	336	0.18	4	1	25
58	237	0.13	2	2	100
**Mean**	**693.7**	**0.35**	**6.3**	**5.1**	**83.4**
**Total**	**12486**		**114**	**91**	

### Confirming generalizability of qualitative findings: Types of collective traumatic grief tweets

Recall that the qualitative fieldwork revealed two distinct types of collective traumatic grief tweets—(1) expressions of acute grief immediately following the loss of a friend, peer, or loved one and (2) expressions of grief on the anniversary of a significant loss or other important dates related to the deceased (e.g., birthdays). Our human-machine partnered approach sought to identify, extract, and analyze tweets in a way such that it would allow us to assess the generalizability of these two modes of the online expression of collective traumatic grief among gang territories/neighborhoods by considering a sample beyond our limited qualitative sample of neighborhoods and respondents. To determine the prevalence of these modes of online expression among the 18 gang territories/neighborhoods in our analytic sample, our domain expert annotators performed a content analysis of the text fields of the individual tweets contained in the 91 spikes in the collective traumatic grief tweets identified earlier as positive matches.

The content analysis determined that a total of 30 of the spikes in the collective traumatic grief tweets (33%) corresponded to acute grief immediately following the death of the subject of an individual. During such spikes, the tweets primarily communicated feelings of intense loss, disbelief, and efforts to self-medicate and occurred usually in the hours and days following the death of the subject of the tweets. For example, the analysis identified that such a spike occurred on July 26, 2014, and that it was associated with Gang 45. The identification and triangulation procedure identified the subject of this moment of community grief as 20-year-old Jevonte “Vonno” Johnson, who was killed the day prior, July 25, 2014, during a failed robbery attempt. The 13 tweets that cause this spike in online grief express a number of intense emotions and mental states. For example, these include the following:

- Woke up tday and my boy gone I’m losing it #[GANG 45] Rip Brosky Von
- [GANG45] Rip Vonno Bro It Hurt So Fuckin Much
- Off hella weed and hen for @[Handle Obscured] rip my boy I love you #[GANG 45] Vonno man this so fucked rest up

Among the elevated moments in the collective expressions of traumatic grief, 35 of the single-daily spikes (38.5%) were classified as those corresponding to anniversaries. These tweets are similarly characterized by intense emotions, although they also contain public calls for local solidarity and collective action. For example, on July, 7 2013, a spike in collective traumatic grief tweets associated with Gang 43 consisted of 6 tweets, all of which corresponded to, and reminded fellow users of, the two-year anniversary of the death of 18-year-old Devonte “Tay” Childress, who was shot on July 6, 2011, and died on July 8, 2011. For example, these tweets included the following:

- I cry Everyday Over [GANG 43] Tay Tay long Live the Legend This shit Crazy smh it Just Don't seem Real
- We On The Block 4 RIP Tayski [GANG 43]

As a further testament to the potential of our human-machine partnered approach to validate and enhance the qualitative analyses, the content analysis determined one additional form of collective traumatic grief expression that had not been previously found in the fieldwork. A total of 26 spikes in collective elevated traumatic grief tweets (28.6%) fell into a residual, or “general”, category, in which tweets corresponded to neither a subject’s date of death nor a subject’s birthday. The tweets associated with these spikes are typically less specific in terms of content or subject. These tweets often mention multiple deceased friends and loved ones and typically lack a discussion of coping strategies and collective action. For example, the following tweet mourns the loss of multiple friends/loved ones by the names “Tutu”, “Crack”, JaJa”, “Johnrynn”, and “Sammy”, while it also expresses love for multiple friends/loved ones who are currently incarcerated:

- [GANG 36] RIPTutu Crack JaJa Johnrynn Sammy lo n all dem

Tweets in this residual category are also likely to mourn the loss of unnamed friends/loved ones. The following tweet is an example of this:

- RIP To All My #[GANG 6] niggas

Although this type of spike may initially appear to be more unpredictable that the previous two types, general moments of elevated trauma are not entirely random. The content analysis found that general moments of elevated trauma are particularly likely to appear when users interact with, consume, and share online content (e.g., songs and music videos) associated with the subject. This is particularly true for users of media sharing platforms, such as YouTube, which broadcast the YouTube users’ activities on Twitter. For example, our fieldwork revealed that before his death on September 4, 2012, Joseph “Lil JoJo” Coleman recorded a song and a homemade music video, which was later uploaded to YouTube by his friends, who added the phrase “RIP JOJO” to the title of the video to honor their deceased friend. Two months after Coleman’s death, on November 11, 2012, an increased number of users interacted with the video. As this activity was subsequently broadcast on their Twitter profiles, it caused a spike in tweets associated with both Coleman and his gang faction. An example of this is the following tweet:

- I added a video to a @YouTube playlist [URL] [Gang 15] RIP JOJO

## Conclusion and discussion

This is the first study that uses a machine-human partnered approach to accurately identify temporal patterns in the online expressions of collective traumatic grief among gang territories/neighborhoods and to verify those online expressions with corresponding offline events. In addition to providing a proof of concept, our results reveal distinctions *between* moments of elevated traumatic grief, which correspond to periods of acute grief, anniversaries related to deceased friends and loved ones, and more general periods of elevated emotions related to ongoing or past traumatic grief. As our analysis demonstrates, these distinct forms of grief- and gang-related Twitter activity are driven by different constellations of offline conditions. Despite the differences between the moments of elevated collective traumatic grief identified in our analysis, all such expressions must be understood as part of a common effort to cope with the serious precarity and insecurities that characterize structurally marginalized neighborhoods.

### Limitations and suggestions for future research

We acknowledge that our analysis of 114 spikes in collective traumatic grief on Twitter represents a minority of the homicides that occurred throughout the study period and that an even greater number of gang- and trauma-related tweets were authored throughout Chicago during our study period. However, one of the driving aims of this study was to provide a proof of concept for our human-machine partnered approach for verifying and increasing the ground truth. In this respect, our analysis proved highly successful. Nevertheless, we identify notable limitations, which can be addressed in future research and applications of our approach. These limitations are largely related to issues of the Twitter search and extraction process, which are hardly unique to this study. First, tweets from deleted accounts are not retrievable through the Twitter Premium API, which retroactively searches through the Twitter stream. There is reason to believe that at least some user accounts and corresponding tweets were deleted between the time of their creation and the time of our search in 2018. The fieldwork indicated that some accounts and tweets are sometimes removed either by Twitter administrators (e.g., for violating Twitter rules) or by users themselves (e.g., deleting content that prompts unwanted attention from rivals, police, etc.). As a result, our analysis may not have incorporated *every* trauma- and gang-related tweet authored during the study period. Future research can overcome such limitations by performing the search and extraction process more frequently (e.g., once per week or even once per hour) to better avoid losing any potential data.

Second, some gang factions’ online identifiers produce relatively more false positives than others. This is most often the case when online identifiers share words, acronyms, and other characteristics with other topics frequently discussed by Twitter users. For example, one gang faction identifies online with an identifier that is similar to a popular nickname for an award-winning musician, Taylor Swift. When Swift’s fans used Twitter to wish her happy birthday, the query and extraction process identified a false positive increase in Twitter activity. We suggest that future researchers carefully assess the noise produced by various identifiers prior to incorporating them into their datasets.

Third, it is important to acknowledge that although the relative frequency of tweets is currently the best available method for identifying elevated moments of collective traumatic grief, incorporating additional measures may provide additional insights. For example, gang-affected communities with fewer than 100 tweets also experience traumatic grief, but they are not included in our approach. Alternative approaches to Twitter will need to be developed to monitor dynamics in traumatic grief for gangs that do not have an online presence. Nevertheless, our method for pinpointing online expressions of traumatic grief is a promising approach that yields a high correspondence to real life violent events.

Fourth, online language is constantly evolving, which means that keywords can become obsolete over time. For example, the use of a particular online gang identifier may decrease as individuals age out, experience incarceration, or leave the neighborhood. In addition, when gang-associated individuals are killed, their names can become new identifiers, supplanting previous identifiers. It is therefore imperative for future researchers to assess the lifespan of particular identifiers and adjust study time frames accordingly. Furthermore, the language surrounding trauma is community specific. For example, our primary trauma keywords (“RIP,” “Rest Up,” and “Long Live”) may not be the most operative terms in communities beyond urban Black and Latinx communities in Chicago. Applying this method to other communities—Latinx neighborhoods in South Los Angeles, for example—will require additional and/or alternative phrases that capture local expressions.

Fifth, our approach does not yet incorporate emojis into the extraction, classification, and analysis workflow. Previous research suggests that emojis may play an important role in the communication of grief, particularly among gang-associated youth [43]. Future research can better assess this hypothesis, and potentially improve the accuracy of our computational techniques, by integrating emojis. To do so, however, will first require qualitative research alongside study populations to catalog the various meanings emojis hold for their online communication.

Finally, all social media platforms undergo periodic changes, which may in turn alter practices of online expression among users. This will influence how future researchers gather data and make sense of linguistic patterns. For example, following our study period, in late 2017, Twitter increased the maximum character limit for individual tweets, from 140 characters to 280 characters. Given the knowledge we gained through our qualitative fieldwork and development of our supervised machine learning classification system, we anticipate that the increase in character limit will allow all users, including those in the communities involved in our current study, to more easily and freely express their emotions and reflect on offline events. We similarly anticipate that the increase will reduce the ambiguity of tweets, thereby making keyword queries and machine learning far more accurate. However, it is ultimately an empirical question as to whether and to what degree this will alter the results of future applications of our approach.

Each of these issues reinforce the importance of integrating community partnerships, immersive fieldwork, and in-depth qualitative interviews into the research design process. Computational approaches to identifying, tracking, and intervening into such complex issues as trauma, gangs, and violence must more centrally involve domain experts, neighborhood residents and, most importantly, those involved in violence (including both offenders and victims). For instance, in our design, the nuanced mixture of trauma- and gang-related vernacular required regular consultations with, and input by, community residents. Research designs that lack such integration carry a high risk of misinterpretation and are likely to have negative consequences for entire communities [38, 41, 54].

### Ethics

It is critical to acknowledge the ethical dilemmas that face current efforts to leverage social media data and computational linguistic analyses for public health research and interventions. Despite their potential for delivering new and innovative forms of trauma-informed services to difficult-to-reach populations, such methods can carry the possibility of harm when deployed by law enforcement and other surveillance institutions. Efforts by law enforcement agencies to use social media data, particular Twitter data, for arrests, prosecutions, and sentencing have already been underway for at least a decade and have targeted communities of color [38, 41]. As the criminalization and surveillance literature increasingly demonstrate, this practice often occurs in the form of “function creep” [55], in which the data collected and analyzed for nonpunitive uses is repurposed for punitive ends [10, 20]. This process exacerbates the criminal justice entanglements of already-marginalized populations, while reinforcing system avoidance and its deleterious effects [20].

Despite these possibilities, we (alongside a growing number of scholars) argue that it is a mistake for researchers and community organizations to simply avoid developing and deploying these technologies altogether [42, 56]. Doing so cedes immense control over these methods to law enforcement and other punitive and surveilling institutions. Instead, it is incumbent on researchers and their community partners to act as an explicit *counterweight* to these punitive uses—to build analyses and tools that are trauma-informed and built explicitly for therapeutic purposes. Thus, rather than turn away from ethical tensions, researchers have a responsibility to directly address these tensions by developing best practices that actively preempt (or, at minimum, seriously mitigate) the possibilities of misappropriation, misinterpretation, and harm that might otherwise result. Our approach does so through four core ethical measures.

First, our trauma-centered approach moves emphasis away from criminality to instead focus on the intense levels of victimization and trauma plaguing structurally disadvantaged communities, as well as the many ways community members experience and process grief collectively. This approach allows us to center the needs of survivors in violence-impacted communities. Rather than conceptualize retaliatory violence as a forgone conclusion, we approach violence as preventable through timely and meaningful support for those who need it most [57]. Second, through our innovative incorporation of qualitative methods, we are better able to ensure that inferences about online activity do, in fact, correspond to offline conditions and events. This also reduces bias in classification and analysis, as well as misinterpretation by future researchers who adopt our approach. Ground truth is further enhanced through iterative consultations with research participants and qualitative data, as well as the close involvement and input by domain experts and local community organizations. Third, we conduct our analysis at the aggregate, community level rather than at the individual level. This approach not only reduces potential risks to individuals involved in our research, it also reduces the possibility that our methods will be used to single out and target individual Twitter users based on their online activity. Fourth, we have undertaken a number of measures to ensure privacy and data security, while retaining research transparency and replicability. This approach entails removing identifying information (e.g., names and Twitter handles) from the data, performing minor alterations in tweet content to render them unsearchable, and only releasing data to researchers who agree to our ethical guidelines precluding them from using the data for punitive purposes. The measures we adopted are among the most stringent used in the field to date. Lastly, we designed our approach and methodology to be optimally useful for community organizations, local stakeholders, and other trauma-informed interventions. We heed Chancellor et al.’s [58] caution to avoid what some refer to as “algorithmic fetishism”—that is, the ongoing drive to develop and use increasingly complex algorithms and other computational techniques simply for the sake of complexity, often at the expense of human understanding. “These algorithms,” warn Chancellor et al. [58], “are ‘black boxes,’ producing impressive results but providing little insight into how the algorithm made its decision” (84). This opacity and lack of accountability alienates relevant stakeholders who wish to adopt these techniques into practical applications.

### Potential intervention applications

Our methods and findings provide a novel opportunity to detect online expressions of traumatic grief that correspond to offline traumatic events, and to intervene in ways that can foster healthy forms of coping, deliver vital services, and reduce retaliatory violence. Our approach provides real utility for community violence prevention organizations, such as Cure Violence, Operation Cease Fire, and Institutes for Nonviolence. These organizations have adopted an epidemiological approach to urban violence that leverages methods traditionally reserved for controlling infectious epidemics (e.g., tuberculosis, cholera, and HIV/AIDS) [57]. To do so, these organizations employ trusted neighborhood residents—often former gang-associated individuals—as outreach workers or “violence interrupters.” When shootings in the local neighborhood occur, outreach workers gather intelligence regarding the identity of the victim, the identity of the offender, and the details of the incident. These workers then reach out to those in the relevant social networks to “interrupt” the transmission of subsequent and often retaliatory violence. The Evaluations of Cure Violence implementation in Chicago showed that shootings dropped by 41%-73% in program communities, and there was a 100% drop in retaliatory homicides in five of eight communities [59].

Our research demonstrates the potential for such organizations to enhance outreach efforts by using Twitter to monitor the moments of elevated traumatic grief in their program areas. Future applications might incorporate automated warnings and mobile “push” notifications to alert organizations when the daily frequency of trauma- and gang-related tweets surpass critical thresholds. For example, by more quickly and definitively identifying spikes in anniversary tweets, local organizations may be able to not only deploy trauma-informed services and support more effectively, but also to potentially anticipate and intercede to prevent violence. Recall that Twitter activity of this type corresponds to offline events and mental states characterized by collective mourning, which often occurs in public gatherings. These offline events may occur in “staging areas” [37], where attendees become targeted by rivals.

Notable instances of offline violence provide empirical support for the idea that events of collective mourning are at elevated risk for further violence. In one of the most publicized examples, on April 12, 2011, the residents of Chicago’s South Shore neighborhood assembled in an empty lot to memorialize the anniversary of the death of local 18-year-old Fazon “Fazo” Robinson. Several of Robinson’s friends, neighbors, and community peers had also been memorializing online, producing a momentary increase in heartfelt tweets about their slain friend. Without warning, members of a rival gang faction opened fire on the crowd, wounding five people, including a 10-year-old boy [60]. By paying closer, more systematic attention to expressions of traumatic tweets in the surrounding area, local South Shore organizations may have potentially been able to intercede and de-escalate such aggressions.

More generally, our research allows for community organizations to acquire information about the emotional climate of the surrounding communities, allowing staff to mobilize and direct support more effectively. Although there were major time investments required to initiate and refine the computational and machine learning component of the research (e.g., qualitative fieldwork, hand-coding), once the model has been trained, it can be applied in near real time and in an automated fashion. The current approach requires that future intervention applications accumulate at least one day’s worth of tweets, which can be compared back to the previous days of Twitter activity for a given gang territory/neighborhood, in order to identify sudden increases in traumatic grief. Although future users may wish to develop a more fine-grained time unit (e.g., hour-by-hour analyses), daily analyses represent a major improvement over what was previously possible.

## Supporting information

S1 Appendix Alternative classification and validation technique. (DOCX)
