# Supplementary material for: A human-machine partnered approach for identifying social media signals of elevated traumatic grief in Chicago gang territories
Source: PLoS One. 2020 Jul 30;15(7):e0236625. doi: 10.1371/journal.pone.0236625 (PMC7392535; doi:10.1371/journal.pone.0236625)
Supplement: S1 Appendix — (DOCX) [file pone.0236625.s001.docx]

S1 Appendix: Alternative Classification and Validation Technique

Using the training data created through hand-coding, we tested a variety of different classification techniques, including support vector machine (SVM) and Multinomial Naive Bayes [1], on the validation set [2]. Naive Bayes and SVM differ in the manner in which they treat and classify the “features” of the text data under scrutiny—in our case, the words contained in tweet text. Naive Bayes classifiers treat features as independent from one another, whereas SVM considers relationships and interactions *between* features. In our application of both classification methods, a positive case is denoted by a tweet that is relevant to the phenomenon of interest. A negative case is denoted by a tweet that is irrelevant to the phenomenon of interest. Using the hand-coded training set as the “true” results, we assessed both models’ prediction of the following outcomes:

1. True positive: the model has correctly predicted a relevant tweet as relevant.
2. True negative: the model has correctly predicted an irrelevant tweet as irrelevant.
3. False positive: the model has predicted an irrelevant tweet as relevant.
4. False negative: the model has predicted a relevant tweet as irrelevant.

In evaluating the classification models, we considered accuracy, precision, and recall calculations as well as the F-score. Accuracy refers to the proportion of tweets correctly identified either as relevant or irrelevant. Precision indicates what percentage of tweets classified as relevant were actually relevant (true positives). If an algorithm does not produce any false positives (i.e., incorrectly classifying an item as relevant), the Precision of that algorithm will be 100% (or 1). Recall is the percentage of actually relevant tweets that are classified as relevant. If an algorithm classifies all the relevant items as relevant, the Recall of that algorithm will be 100% (or 1). The F-score is the harmonic mean of precision and recall and is defined as follows: F-score=2*R*P/(R+P), where R indicates Recall, and P indicates Precision [3].

The best performing classifier was found to be the Naive Bayes which, based on our empirical tests, yielded an accuracy score of 84% on the validation set [50]. Table 1 presents the results of comparing two classification methods for the tweets with the “RIP” keyword.

**Table 1. Results of Naive Bayes and SVM Classifiers on the Validation Set.**

|  | **Accuracy** | **Precision** | **Recall** | **F Score** |
| --- | --- | --- | --- | --- |
| Naive Bayes | 0.84 | 0.7 | 0.82 | 0.75 |
| SVM | 0.83 | 0.74 | 0.66 | 0.7 |

Next, to further evaluate the effectiveness of our chosen classification model, we applied the Naive Bayes classifier on a randomly selected sample of 200 preprocessed tweets (100 relevant and 100 irrelevant, as indicated by the classifier algorithm). Again, drawing on fieldwork, subject knowledge, and coding rules, our two domain experts hand-coded each tweet in the sample as relevant or irrelevant. Finally, we compared the hand-coding results to the results predicted by our Naive Bayes classifier model. We present the results in Table 2. Our chosen Naive Bayes classifier performed well, with an F-score of 0.76. The keyword-based approach we present here is a first proof-of-concept. Because of its simplicity, this approach lends itself to application in community settings. To assess improvements in classification, future studies could experiment with additional computational resources, such as deep learning.

**Table 2. Results of Naive Bayes Classifier on the Validation Set.**

| **Accuracy** | **Precision** | **Recall** | **F Score** |
| --- | --- | --- | --- |
| 0.725 | 0.78 | 0.75 | 0.76 |

References

1. Lewis DD. Naive (Bayes) at forty: The independence assumption in information retrieval. In: Nedellec C, Rouveirol C, editors. Proceedings of ECML-98, 10th European conference on machine learning. Heidelberg: Springer; 1998. pp. 4-15.

2. Cortes C, Vapnik V. Support-vector networks. Mach Learn. 1995;20: 273-297.

3. Sasaki Y. The truth of the F-measure. Teach Tutor Mater. 2007;1: 1-5.
